# Advances in the immunoescape mechanisms exploited by alphaherpesviruses

**DOI:** 10.3389/fmicb.2024.1392814

**Published:** 2024-06-19

**Authors:** Yimin Wang, Caoyuan Ma, Shan Wang, Hongxia Wu, Xuanqi Chen, Jinyou Ma, Lei Wang, Hua-Ji Qiu, Yuan Sun

**Affiliations:** ^1^Henan Institute of Science and Technology, Xinxiang, China; ^2^Ministry of Education Key Laboratory for Animal Pathogens and Biosafety, Zhengzhou, China; ^3^State Key Laboratory for Animal Disease Control and Prevention, Harbin Veterinary Research Institute, Chinese Academy of Agricultural Sciences, Harbin, China

**Keywords:** alphaherpesviruses, immunoescape, immune regulatory proteins, latency, signaling pathways

## Abstract

Alphaherpesviruses, categorized as viruses with linear DNA composed of two complementary strands, can potentially to induce diseases in both humans and animals as pathogens. Mature viral particles comprise of a core, capsid, tegument, and envelope. While herpesvirus infection can elicit robust immune and inflammatory reactions in the host, its persistence stems from its prolonged interaction with the host, fostering a diverse array of immunoescape mechanisms. In recent years, significant advancements have been achieved in comprehending the immunoescape tactics employed by alphaherpesviruses, including pseudorabies virus (PRV), herpes simplex virus (HSV), varicella-zoster virus (VZV), feline herpesvirus (FeHV), equine herpesvirus (EHV), and caprine herpesvirus type I (CpHV-1). Researchers have unveiled the intricate adaptive mechanisms existing between viruses and their natural hosts. This review endeavors to illuminate the research advancements concerning the immunoescape mechanisms of alphaherpesviruses by delineating the pertinent proteins and genes involved in virus immunity. It aims to furnish valuable insights for further research on related mechanisms and vaccine development, ultimately contributing to virus control and containment efforts.

## 1 Introduction

Herpesviruses comprise a collection of viruses possessing a genome of double-stranded DNA, which are traditionally believed to be stable and uniform in term of genetic variabilities (Kuny and Szpara, 2021). The complete viral architecture of the herpesvirus encompasses distinct layers, namely the genetic material, capsid, tegument, and envelope, arranged sequentially from the innermost to the outermost layers (Baines, 2011). According to the cell types responsible for initiating infection and the length of their replication cycles, herpesviruses are categorized into three subfamilies, *Alphaherpesvirinae*, *Betaherpesvirinae*, and *Gammaherpesvirinae* (Sehrawat et al., 2018). *Alphaherpesvirinae* exhibit the widest variety of hosts, undergo rapid replication, leading to cytopathic effects during lytic infection, and enter a latent state within the sensory ganglia. The majority of alphaherpesviruses sustain persistent latency within neurons, with exceptions found in non-neuronal tissues, such as Marek’s disease virus (MDV) or gallid alphaherpesvirus type 2 (Bloom, 2016). *Betaherpesvirinae* exhibit the narrowest host spectrum and a relatively sluggish replication pace, frequently leading to cell enlargement (giant cell proliferation), and it enters a latent state in diverse anatomical sites and cellular populations, encompassing secretory glands, kidneys, and lymphoid reticulum cells. *Gammaherpesvirinae* invade lymphoblast-like cells and commonly demonstrate selectivity towards either T or B lymphocytes, leading to the establishment of a latent phase within lymphoid tissues (Pomeranz et al., 2005).

Alphaherpesviruses exhibit a biphasic life cycle that closely mirrors the clinical progression in most individuals. They encompass two distinct phases: acute infection, and latency (White et al., 2012). The acute phase is typically either asymptomatic or characterized by mild rash and fever symptoms. Following the acute phase, the virus enters a latent phase with no noticeable disease symptoms present in the host. The latent phase is defined by the suppression of lytic gene expression and the circularization of the genome (Aschner and Herold, 2021). Intermittently, latent genomes are reactivated, giving rise to progeny virions, which migrate along nerve fibers to the initial site of infection. There, the viruses may undergo local replication within the mucosal epithelium and subsequently be shed. The hallmark of clinical reactivation is the vigorous re-emergence of viral genetic activity and replication, leading to significant inflammation and damage to tissues (Ostler and Jones, 2023).

Immunoescape refers to the viral capacity to evade or overcome the immune response of the host. During the initial invasion, the body’s defense mechanisms identify and target viruses, but some viruses have developed strategies to evade detection and destruction (Nelson et al., 2020). In the context of herpesviruses and other persistent viruses, immunoescape mechanisms can allow the virus to persist in the body and establish a latent infection (Singh and Tscharke, 2020). The virus enters a latent state during a dormant phase, residing in certain cells without causing active disease. The immune system may not effectively recognize or eliminate the latent virus due to the strategies employed by the virus to immunoescape surveillance. This successful evasion results in the onset of disease and involves tactics such as altering surface antigens, impeding the recognition by host immune cells, mimicking host cells to elude immune surveillance, and suppressing the host immune responses (Penfold et al., 1994). As individuals becomes old or experiences changes in their immune function, the equilibrium between the immune response and the virus is disrupted. This could result in the awakening of the latent virus, leading to the reappearance of active infection and potential transmission to other hosts (Mazziotta et al., 2021). Thus, immunoescape can contribute to the virus persisting in establishing latent infections within the body (Flori et al., 2008).

Herpesviral infections, owing to their immunoescape capabilities, represent a considerable challenge to both individual health outcomes and public health (Ling et al., 2003). While severe diseases caused by herpesviruses are in frequent, the presence of multiple types of herpesviruses in most adults’ bodies becomes a concern when encountering immunocompromised individuals, leading to potential serious threats (Chen et al., 2022). The initial replication of PRV takes place on the mucous membranes of the nasal cavity and oropharynx. PRV targets the respiratory and nervous system tissues of pigs, entering sensory nerve terminals within the infected mucosal epithelium (Pan et al., 2023). In the acute stage of infection, viral particles replicate in the oral and pharyngeal mucosa, subsequently evading immunity by entering sensory nerve endings in the affected area (Li et al., 2022). Retrograde conveyance of viral particles takes place in the maxillary branches of the trigeminal, glossopharyngeal, and olfactory nerves, establishing latency. Consequently, the purification path of PRV is fraught with difficulties (Maes et al., 1997). In the context of human herpesviruses, the varicella-zoster virus (VZV) as an example. A unique feature of VZV is its exclusive infection of humans with no known animal host. VZV targets T lymphocytes, epithelial cells, and nerve cells (Kennedy and Gershon, 2018). During the initial infection, chickenpox becomes apparent, and VZV establishes a latent infection in neuronal ganglia by employing immunoescape mechanisms (Kennedy, 2016). As elders’ immune system becomes compromised, the cell-mediated immunity to VZV is attenuated, facilitating VZV reactivation (Kennedy et al., 1998). This phenomenon commonly manifests as herpes zoster, which can give rise to various complications, including postherpetic neuralgia (PHN) and a spectrum of other associated health issues (Gershon et al., 2015).

Since alphaherpesviruses such as PRV is a potential threat to public health and economic development, given that the virus can remain latent for life after infecting the host, exploring the mechanism of immunoescape is crucial for the elimination of alphaherpesviruses. This review primarily elucidates the establishment and reactivation of alphaherpesvirus latency, focusing on key viral infection proteins. It further delves into recent discoveries regarding immunoescape mechanisms utilized by alphaherpesviruses during latency, aiming to gather pertinent insights while establishing a theoretical framework for comprehending the pathogenic mechanisms of alphaherpesviruses. Ultimately, it seeks to furnish valuable information to aid in the development of potential vaccines.

## 2 Establishment of latency and reactivation of alphaherpesviruses

### 2.1 Establishment of latency by alphaherpesviruses

The incubation period refers to the time before the herpes virus becomes active (Doll et al., 2020). Researchers used inhibitors to prevent the replication of HSV-1 and infected mice with replication-deficient viruses, both of which successfully induced the virus’s incubation period (You et al., 2021). Further investigations revealed that ICP4 viruses only yield low levels of the *IE* genes, and any viruses with a deficiency in *IE* function can effectively establish a latent period. This suggests that the establishment of a latent period may not necessarily depend on gene expression cleavage.

**Figure 1:**
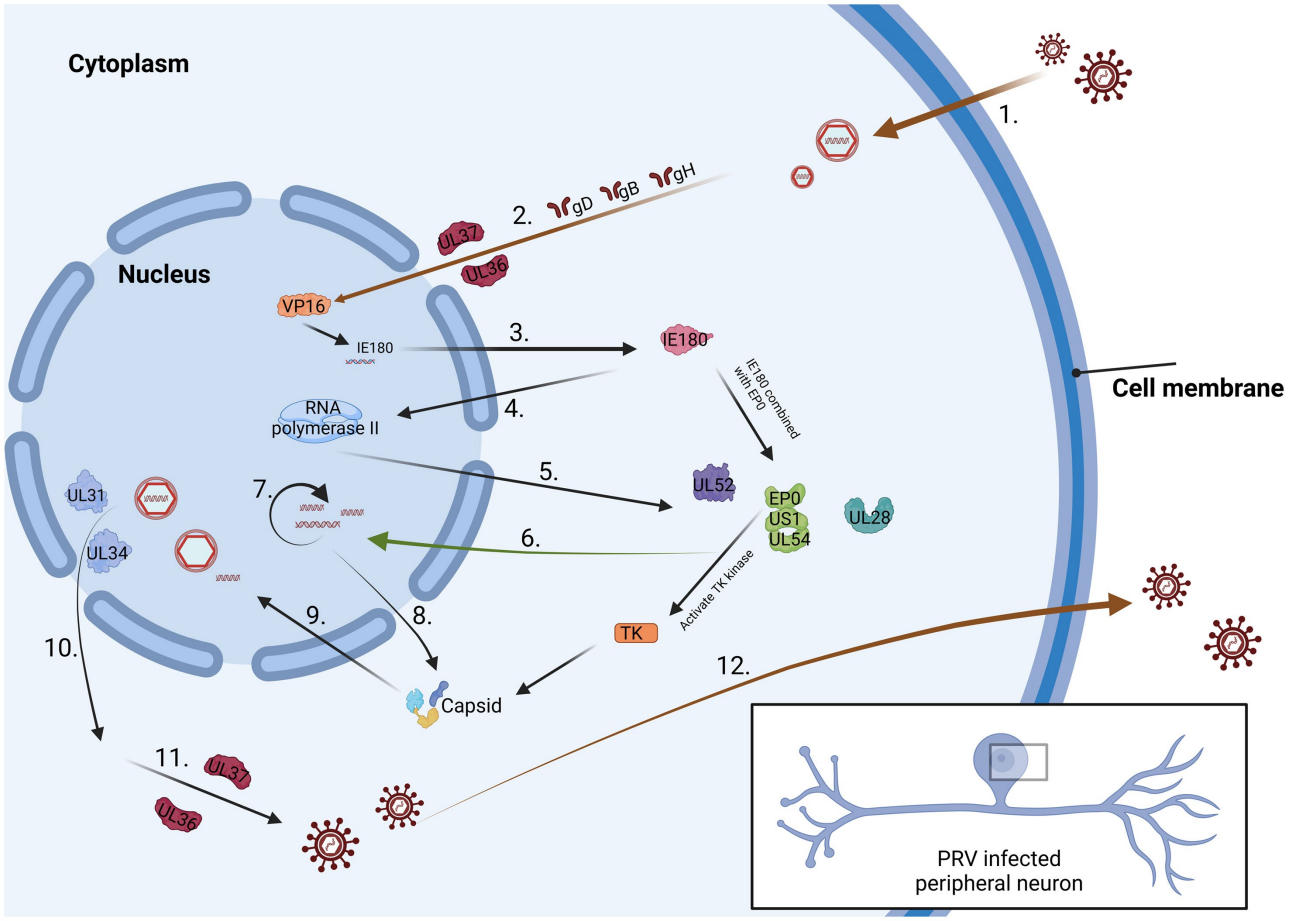
The process of PRV infection of neurons. 1. Virus surface proteins attach to the cell membrane. 2. The virus passes through the cytoplasm and reaches the nucleus 3. IE180 is activated by VP16 protein and expressed in the cytoplasm. 4. IE180 returns to the nucleus and activate RNA polymerase II. 5. Viral proteins are produced within the cytoplasm. 6. These proteins can promote viral DNA replication. 7. Viral DNA synthesis is considered a late stage in the PRV replication cycle. 8. Capsid protein is synthesized in the cytoplasm. 9. The protein is sent back to the nucleus and preliminarily packaged. 10. The fully assembled virus leaves the nucleus. 11. The virus enters the trans-Golgi apparatus by binding to the envelope and envelope proteins. 12. The mature virus is conveyed to the cellular surface.

At the cellular level, sensory nerve cells within the peripheral nervous system form a varied and exceptionally specialized group of cells. Currently, nearly two dozen characteristics specific to sensory neurons have been identified, and they are present in sensory ganglia among individuals (Marshall et al., 2019). Specialized ganglia, such as the trigeminal ganglion (TG), exhibit distinct manifestations. Notably, sensory neurons represent the primary site where alphaherpesviruses establish their latency period, exemplified by PRV and HSV-1 (Figure 1). For PRV entering a latent infection state, its genome predominantly resides in neurons of the TG (Zhou et al., 2023). During this phase, the activity of viral lytic genes is entirely inhibited, and transcription is restricted to a compact area recognized as the latency-associated transcript (LAT) locus. From the perspective of cellular immunity, HSV-1 has been noted to initiate latent infection within distinct sets of sensory neurons in the trigeminal ganglion. Studies employing antibodies directed against functional receptors, like the high-affinity nerve growth factor receptor trκA, molecules associated with pain perception (substance P receptors), or surface markers expressed by different subsets of sensory neurons, have yielded valuable insights. Analysis of these findings suggests that HSV-1 mainly establishes latency within a subset of trκA^+^ neurons expressing surface molecules identified by the monoclonal antibody A5 (Bertke et al., 2011). On the contrary, HSV-1 typically initiates productive infections within neurons that express cell surface molecules recognized by the monoclonal antibody KH10. The molecular mechanisms that differentiate neurons, supporting either productive or latent infection, remain unclear. However, this observation underscores the involvement of sensory neurons within the complex biology of alphaherpesviral latency. It is noteworthy that the establishment of the incubation period aligns with the acute infection stage (Kapadia et al., 2002). In essence, sensory nerve cells are pivotal in initiating the latent phase of alphaherpesvirus, with their differentiation status and specific surface marker expression patterns significantly influencing both viral latency and productive infection.

Latent infection is the result of the coordinated regulation of neurons, viruses, and immune system (Szpara et al., 2010). When the delicate equilibrium is disturbed, the virus can establish latency within the host. At this point, the viral genome successfully hides within the nucleus, but gene expression is restricted, and the process of virus replication is paused (Banks and Rouse, 1992). In that way, the latency is established, which means host cannot clear the virus in life span. From the perspective of the viral genome, the first step involves releasing it into the nucleus of neurons through nuclear pores, where the transcription and translation functions of most genes are limited. Subsequently, the virus extends the survival duration of the infected LAT cells, imparting anti-apoptotic effects and enabling evasion of host immunity. This mechanism serves to safeguard potential genomes (Zhang et al., 2021; Zhang and Tang, 2021). In the end, dormant viruses can monitor and influence the condition of host cells, waiting for the opportune moment to undergo reactivation (Deng et al., 2022). The interaction between the virus and host cells ultimately safeguards the viral genome and facilitates the reactivation of the virus during its latency.

Latent infection can be interpreted as a host defense mechanism against viral fragmentation programs, leading to virus silencing and halting the progression of the disease. Alternatively, the incubation period may be seen as the virus lying dormant until an immunocompromised population emerges, thereby facilitating additional virus transmission. A prerequisite for establishing a latent infection method is that the virus genome must maintain transcriptional silencing in regions that can sense the state of host neurons, thereby evading immune clearance (Capozza et al., 2021). Studies have demonstrated significant distinctions between the course of the initial lytic infection and the processes regulating viral reactivation after entry into latency. These differences may be associated with the establishment of viral latency.

### 2.2 Research on the mechanism of reactivation

The study revealed the presence of two primary forms of activation: spontaneous reactivation and induced reactivation. Spontaneous reactivation primarily characterizes low-level detectable reactivation. Induced reactivation predominantly ensues following the application of chemical or physiological agonists, leading to bursts of reactivation. A noteworthy characteristic of induced reactivation is its capacity to markedly enhance virus shedding shortly after initiation (Smith et al., 2004). Beyond augmenting the frequency of reactivation, induction generally elicits a greater array of clinical symptoms compared to the typical manifestations associated with spontaneous reactivation (Miranda-Saksena et al., 2000).

In summary, research from animal models suggests that the reactivation of HSV-1 leads to the release of detectable infectious viruses in the peripheral area, often occurring without clinical lesions (Nicoll and Efstathiou, 2013). Introducing external stressors can induce reactivation events that are more predictable and intense, thereby increasing the likelihood of their manifestation as clinical lesions. The comparative assessment of deliberate and natural reactivation in animal models unveils a significant correlation between physiological and emotional stressors and reactivation in humans (Roizman and Whitley, 2013).

Upon reactivation, the virus reproduces in neurons and is transported forward to the original entry point or a nearby location capable of entering the body. Studies have identified various factors influencing the nature and reactivation rate of latent viruses, encompassing the virus type, host genetics, and the surrounding environment (Yu et al., 2013).

The involvement of viral genetic material in the reactivation process is primarily examined from two perspectives. There is indeed a correlation between the viral load and the frequency and severity of recurrent lesions (Salazar et al., 2023). In a mouse model, both the quantity of viruses and the frequency of lesions are contingent upon the amount of virus present at the time of vaccination. In cases of human genital HSV-2 infection, it is postulated that the severity of the initial infection is indicative of the viral load at that time, emerging as a significant factor linked to the intensity of subsequent recurrences. The intensity of the primary infection may, in turn, be indicative of the host’s susceptibility (Tronstein et al., 2011). The activation of the alphaherpesvirus primarily occurs through two mechanisms, with induced reactivation typically instigated by chemical or physiological agonists. Additionally, external stressors may heighten the likelihood of reactivation, while both viral load and genetic factors influence the frequency and severity of such occurrences.

## 3 The models for studying latent infections of alphaherpesviruses

**Figure 2:**
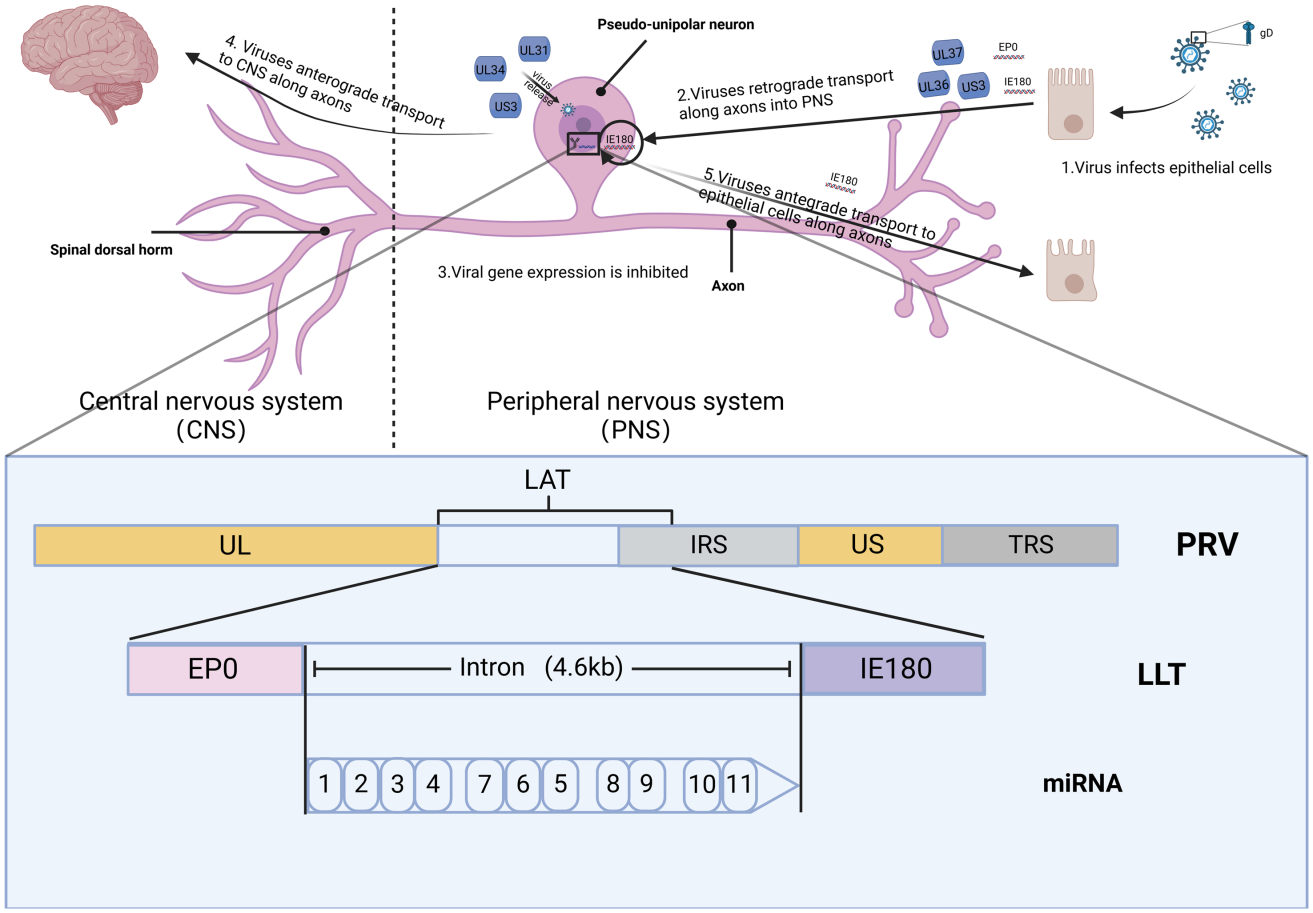
The spread of PRV within the nervous system. The figures for this study can be found in the biorender (BioRender.com).

Alphaherpesviruses establish latency within the peripheral nervous system (PNS) of the host, ensuring their prolonged persistence in the host population (Figure 2). Researchers elucidate the distinct roles of PRV in latent infection and neural conduction at the genetic level. The genomes of eukaryotes typically feature a singular initiation site for DNA synthesis, referred to as the origin of replication (*ori*). In contrast, viruses possess one or multiple *oris*, determined by the structural characteristics and sequence specificity of particular DNA fragments. In HSV-1, there are three oris present: two located within the inverted repeats (designated as *oris*) surrounding the unique short (US) region, and one within the unique long (UL) region (referred to as *oril*). For PRV, *oril* is situated in the intergenic region between the *UL21* and *UL22* gene pairs. These genes play a crucial role in DNA replication. Increasing evidence suggests that certain non-coding transcripts, including short non-coding RNAs (ncRNAs) such as microRNAs (miRNAs) and long ncRNAs (lncRNAs), play significant roles in regulating DNA replication. During the virus incubation period, LAT, as the only highly expressed gene sequence, has been found to regulate DNA replication through 12 identified non-coding RNAs. Various modes of controlling DNA replication exist. This includes the regulation of RNA primer synthesis through hybridization with DNA sequences or forming hybrids with mRNA. This hybridization process initiates their degradation through RNase H, thereby inhibiting replication and protein translation (Torma et al., 2023). In VZV, aside from LAT and ORF63, no other VZV mRNA expression was detected during infection. A notable correlation between LAT and ORF63 suggests their joint regulation of expression during VZV latent infection. This expression of two distinct viral transcripts during incubation is a well-studied phenomenon unique to alphaherpesviruses, suggesting that these transcripts and their encoded proteins likely play crucial roles in VZV latency and activation. The encoded pVLT-ORF63 fusion protein is implicated as a promoter for VZV reactivation in infected human TG, offering novel insights into latent and reactivation mechanisms for VZV. Further research is warranted to ascertain the neuronal types of VLT and ORF63 RNA, along with their association with the latent VZV genome. In contrast, there are no reported promoters in HSV-1 driving viral gene expression during reactivation, indicating potential differences in the mechanisms governing latent infection and reactivation between HSV and VZV (Wu et al., 2021).

Studying neurons infected with PRV has advanced our comprehension of viral assembly and long-distance transport within axons (Adamou et al., 2023). Research indicates that the mechanism through which viruses enter neurons closely parallels that observed in cultured cells. Initially, the viral envelope merges with the membranes of healthy epithelial cells located at the axon terminals of the peripheral nerve endings in the natural host (Wilson, 2022). Following viral particles’ entry into cells through membrane fusion, a process of matrix protein depolymerization ensues, leading to the majority of viral matrix proteins entering the cytoplasm. Upon entry into the cell, specific matrix proteins, including UL14, ICP0, US3, UL36, and UL37 persistently remain attached to the capsid (He et al., 1997). The capsid, accompanied by the inner membrane proteins, is retrogradely transported along the axon for a substantial distance, ultimately reaching the nucleus of the sensory neuron (He et al., 2019). Research indicates that viral capsids undergo swift retrograde transport in axons. During the transport into axons, the capsid is exclusively associated with certain membrane proteins, namely UL36 and UL37, while other proteins such as UL47, UL48, and UL49 seem to be unrelated to this process or do not have associated effects (Workman et al., 1988). UL37 is crucial in regulating the early viral *IE180* expression and facilitating the retrograde conduction of the virus within the axon following the entry of virus particles into neurons (Kramer et al., 2011). UL36 serves multiple crucial functions, including transporting capsids to the nucleus, facilitating the docking of capsids with nuclear pores, and releasing viral genomes within the nucleus (Murray and Wynn, 2011). The hypothesis suggests that the movement of viral capsids within axons relies on molecular motors, probably from the dynein family. If the capsid were to rely solely on diffusional movement to reach the neuronal soma, it would be anticipated to take several years for this process to occur (Rziha et al., 1986). After invading neurons, the virus undergoes the depolymerization of matrix proteins, retrograde conduction along axons, docking of capsids with nuclear pores, and ultimately delivers the viral genome within the nucleus. Following entry into the nucleus, the linear viral genome initiates a process of circularization, wherein the circular DNA acts as a template for viral genome replication. In the case of PRV, gene transcription is initiated by the expression of the immediate early gene *IE180* (Van Opdenbosch et al., 2012). The freshly replicated viral genome undergoes packaging into a capsid within nucleus. This process entails the polyDNA formed during replication being cleaved into monomeric linear genomes, which are subsequently packaged into capsids. After encapsulation, PRV nucleocapsids travel from the nucleus through the nuclear membrane and enter the cytoplasm (Reynolds et al., 2002). The export of herpesvirus nucleocapsids relies on the viral proteins US3, UL31 and UL34 (Gant Luxton et al., 2005). Nevertheless, recent research has suggested that additional viral matrix proteins and nuclear envelope glycoproteins are linked to the initial acquisition of the envelope by virions. However, the specific role of these proteins in the nuclear exit process remains unclear. Reactivation from latency entails the activation of transcription, the assembly of mature virus particles, and the transmission of contagion from the neuronal cell body through axons back to surrounding epithelial tissues (Heming et al., 2017). As capsids move in the direction of the nucleus upon entry and towards the axon terminals upon exit, a mechanism must exist to control the orientation of capsid transport throughout infection. The orientation of transport is dictated by the motor complex associated with the capsid: retrograde transport is facilitated by motor proteins, whereas kinesin proteins support anterograde transport. In a study by Smith, freshly produced green fluorescent protein (GFP)-tagged capsids were observed to traverse monomeric red fluorescent protein-tagged capsids in opposite directions within the same axon (Smith et al., 2004). Therefore, the regulation of transport direction and the resulting coordination of movement occur at the level of individual capsids rather than through the overarching control of axonal transport (Wang et al., 2020).

Most alphaherpesviruses follow a common infection pathway: they first initiate a primary infection on mucosal or epithelial surfaces and subsequently migrate to the PNS, where they establish latent infections that can be reactivated (Wang et al., 2022). Infection of PNS neurons hosts often results in an inactive and latent viral condition (Diefenbach et al., 2008). This revelation underscores the significant influence of host cell characteristics on the development of either productive or latent infections. Furthermore, host factors may also play a role, as various external stressors can trigger the reactivation of the latent phase (Ch'ng and Enquist, 2005).

As an example, considering HSV-1, it can swiftly cause damage on non-neuronal cells and establish residence in ganglionic neurons. This is achieved through the formation of a protein complex comprising the HSV-1 VP16, along with cellular proteins such as HCF-1, Oct1, LSD1, and a histone acetyltransferase known as CLOCK. In the nucleus, the VP16 protein complex functions in histone demethylation, facilitating the transcription of the HSV-1 *α* gene. However, in neurons, these processes encounter a blockade, preventing the transfer of VP16 and HCF-1 to the neuronal nucleus (Thompson et al., 2009). As a result, the resident nuclear protein complex, comprising HDAC/CoREST/LSD1/REST, remains active and promotes post-translational modifications of histones bound to the incoming nuclear virus DNA, ultimately leading to gene silencing (Gaidt et al., 2021). In a murine model, the majority of CD8^+^ T cells within the latently infected ganglia target a specific seven-amino-acid linear epitope on the HSV-1 glycoprotein B (gB), while the remaining T cells target HSV-1 proteins directly and possess the ability to inhibit virus reactivation. In contrast to HSV-1, VZV replicates rapidly and efficiently, generating a syncytium composed of neurons and satellite cells, and then releasing viruses. Within neurons that manage to survive, minute quantities of VZV DNA and RNA can be identified, even without discernible viral proteins (Suvas and Rouse, 2006). Mutation analysis of the infecting virus underscores the critical role of the interaction between the gI and gE of VZV in viral transmission within xenografts (Cohrs and Gilden, 2011).

Feline herpesvirus type 1 (FeHV-1) undergoes a latent phase following the acute stage, with reactivation evidenced by the recurrence of clinical symptoms (Gaskell et al., 2007). Latent infections have been confirmed in the TG, where FeHV-1 DNA has been detected along with other sites, indicating viral genome presence. However, detecting latency-associated transcripts (LATs) remains the most reliable method to ascertain true latency (Ferrara et al., 2023). In FeHV-1, LATs have been identified in the trigeminal ganglia through *in situ* hybridization. While mounting evidence suggests an association between FeHV-1 and chronic eye diseases, akin to herpes simplex virus, it’s noteworthy that while FeHV-1 DNA was found in both trigeminal ganglia and cornea, LAT transcripts were solely detected in the former, implying a potential absence of true latency in the cat corneal membrane (Monne Rodriguez et al., 2017). Consequently, further investigation into gene transcription and expression in latently infected tissues is warranted.

Consistent with other alphaherpesviruses, EHV-1 exhibits an incubation period in both lymphoid and neural tissues. CD5^+^/CD8^+^ T lymphocytes are identified as the primary site of EHV-1 latency, activated independently or indirectly by IL-2 and equine chorionic gonadotropin. EHV-1 can infect various cell types, including endothelial cells in internal organs, respiratory epithelial cells, lymphoid organs, and monocytes in peripheral blood, either through direct contact with infectious particles or intercellular transmission. Following initial infection, EHV-1 can persist in susceptible host cells for life. During the initial stage of EHV-1 infection in the upper respiratory tract epithelium, a latent period is established, during which infected horses remain asymptomatic with no clinical signs, viral shedding, or cell associated viremia (Hussey and Giessler, 2022). The primary site of EHV-1 latency established in horses remains controversial. Some studies suggest it occurs in circulating lymphocytes and lymphoid cells in draining lymph nodes, while others propose sensory neuron bodies in the trigeminal ganglion as the preferred primary site. Regardless of the site, EHV-1’s ability to enter latency appears to be a deliberate biological behavior facilitating its perpetuation within the host, enabling spread upon reactivation (Jones et al., 2006). During latency, EHV-1 genome expression is suppressed, with only LATs present, antisense to immediate early viral genes or regulatory early genes in infected cells (Patel and Heldens, 2005).

CpHV-1 can establish latent infections in the trigeminal ganglia and inducing immune suppression, similar to HSV-1 and PRV of the *Alphaherpesvirinae* subfamily. CpHV-1 establishes a lifelong, non-productive latent infection in ganglionic sensory neurons. Following herpesvirus infection, certain neurons become effectively infected during the acute phase, while others resist viral replication, leading to latency (Forte et al., 2021). Throughout the incubation period post-acute phase, gene expression is restricted to a specific genomic region known as the latency-related (LR) region. Extensive transcription of the *LR* genes, including the open reading framework-E (ORF-E), serves as a marker of latency. ORF-E, downstream of the *LR* genes, encodes the main viral transcription activator b ICP0 (Lanave et al., 2020). Various factors contribute to incubation period establishment post-acute infection, including the *LR* genes in TG, ORF-E, neuronal factors, and cell-mediated immune (CMI) responses. The *LR* gene products prevent apoptosis in virus-infected cells, thereby inducing latency and viral gene expression (Montagnaro et al., 2013). ORF-E potentially aids in latency establishment by promoting neuronal dendritic growth in mouse neuroblastoma cells, potentially facilitating mature neuronal function recovery post-infection. Neuroeffectors inhibit productive infection and TG CMI responses, leading to extensive viral transcripts, proteins, and DNA replication, thereby inducing latent infections (Camero et al., 2010).

In general, although all alphaherpesviruses undergo latency in neurons, the incubation period of HSV-1 is restricted to the cerebral ganglia, while HSV-2 remains latent in the sacral ganglia (Depledge et al., 2018). The distinction lies in the fact that VZV reside in multiple ganglia spanning the entire neural axis, engaging in transcription of multiple viral genes, and revealing specific viral proteins (Depledge et al., 2018). Moreover, in ganglia with latent HSV-1 infection, specialized T cells specific to the virus and virus-encoded miRNAs work together to impede the reactivation of HSV-1 (Szpara et al., 2010).

The alphaherpesvirus establishes latency within the host’s nervous system, undergoing long-distance transport via axons to reach neuronal nuclei, where it replicates its genome and releases it into the cytoplasm. Latent infections typically manifest on mucosal or epithelial surfaces and can be reactivated by external stimuli. HSV-1 and HSV-2 remain dormant in the brain and sacral ganglia, respectively, with specific T cells and microRNAs playing crucial roles in preventing HSV-1 reactivation. While previous research has largely emphasized processes such as infection and replication in simplex neuron cells and other cell types, our perspective differs to that, researchers consider all cells to be interconnected within a complex system. Consequently, our emphasis lies on the diverse elements of the immune system (Qi et al., 2019).

## 4 Introduction to immunoescape mechanisms

At present, it is understood that the alpha herpesvirus employs diverse strategies to elude immune surveillance. These strategies encompass infecting tissues with restricted access to immune mediators, enabling the virus to establish a latent period and evade minimal immune recognition. Concurrently, the alphaherpesvirus orchestrates several active immune regulatory programs (Peng et al., 2009). To enhance the understanding of the immunoescape mechanisms, the focus lies on examining the products generated during the viral immunoescape process.

### 4.1 gC

The complement-modified proteins widely present in pathogenic viruses, and their significant roles in disease progression render them potential targets for preventive or therapeutic strategies (Sathiyamoorthy et al., 2017). Remarkably, PRV, HSV-1, and other alphaherpesviruses demonstrate the capacity to suppress complement activation, a phenomenon closely linked to gC. Concerning HSV-1, gC interacts with complement component C3, hindering complement-mediated virus neutralization and *in vitro* cell lysis (Lubinski et al., 1998). This inhibition extends to triggering the complement cascade, disrupting the classical complement pathway, and bypassing the amplification of the complement pathway along with the production of attack membrane complexes (Connolly et al., 2021). Extensive evidence supports the assertion that HSV-1 gC orchestrates diverse activities *in vitro*, including complement-mediated acellular virus neutralization and the inhibition of infected cell lysis through complement mediation (Stock et al., 2019). The efficacy of gC in averting complement-mediated inactivation of wild-type viruses is noteworthy (Ren et al., 2020). Investigations involving C3 knockout mice revealed negligible differences between complement-intact wild-type viruses and C3D animals. In C3D guinea pigs, the wild-type titer only exhibited a marginal increase compared to animals with an intact complement (Rizvi and Raghavan, 2003). These findings align with *in vitro* experimental outcomes, indicating minimal neutralizing effects of complement on wild-type viruses or lytic effects on infected cells (Li et al., 2022). Researchers deduce that gC affords nearly complete protection for wild-type viruses during complement attacks. This observation suggests that gC can impede antibody-independent complement activation, corroborating *in vitro* findings (Yamada and Shimizu, 1994). While this protective role is pivotal for virus-infected cells, the specific complement pathway modulating HSV infection *in vivo* remains a topic required further exploration (Guo et al., 2010). Compared with HSV gC, VZV gC has a similar effect. It binds to IFN-*γ* and alters its activity. After combined, it increased the expression of a small portion of IFN-stimulating genes (ISGs), including intercellular adhesion molecule 1 (ICAM1), as well as some chemokines and immune regulatory genes (Kennedy et al., 2015). During the infection process, the presence of gC increases the efficiency of VZV diffusion. And with IFN-*γ* Conduct stable interactions through IFN-*γ* the receptor emits signals, which is a very important inspiration for the functional research of gC proteins (Kennedy et al., 2021). Overall, complement C3 plays a pivotal role in humoral immunity, acting as a crucial link between innate and adaptive immunity. The gC protein exerts its influence by inhibiting complement-mediated viral neutralization and cell lysis reactions through interaction with complement molecule C3 in humoral immunity. This mechanism aids the virus in evading immune surveillance and clearance by host cells, thereby facilitating virus survival and transmission. Hence, the function of the gC protein is intricately intertwined with immunity, as it fosters viral infection and survival by interfering with the host cell’s immune response.

### 4.2 ICP47

Some herpesviruses express a 9-kDa product of the * IE* gene product known as ICP47, which acts as a soluble inhibitor of the TAP complex, impeding peptide transport. TAP is a protein complex essential for peptide transportation from the cytoplasm to the endoplasmic reticulum (ER), playing a crucial role in antigen presentation on the cell surface—an indispensable step in the immune response (Wilson, 2021). The viral immunoescape strategy involves ICP47 blocking peptide binding by interacting with TAP1 and TAP2 on the cytoplasmic face of the endoplasmic reticulum (Tronstein et al., 2011). Notably, segments 2–35 of ICP47 constitute the minimal regions necessary for interaction with TAP and the hindrance of peptide transport. ICP47 accomplishes this by diminishing the linkage between TAP1 and TAP2. It is noteworthy that ICP47 exhibits considerable species specificity. It can inhibit TAP in humans, pigs, and dogs; however, it does not affect TAP in mice, guinea pigs, and rabbits. TAP is a vital component of cellular immunity, playing a pivotal role in antigen presentation. In cellular immune responses, antigen-presenting cells (such as dendritic cells, macrophages, and B cells) present endogenous antigen fragments (such as proteins produced by viral infections or tumor cells) to specific immune cells, namely CD8^+^ T cells (also known as cytotoxic T cells), via major histocompatibility complex (MHC) class I molecules. This process is critical for initiating and regulating specific cellular immune responses. ICP47 executes the virus’s immune escape strategy by interacting with TAP1 and TAP2 on the cytoplasmic surface of the endoplasmic reticulum, thereby impeding peptide binding.

### 4.3 ICP34.5 (US11)

In the context of HSV-1, the gene is often called the “*γ34.5* gene” or “*ICP34.5* gene.” The *γ34.5* gene encodes a protein known as infected cell protein 34.5 (ICP34.5). When stimulated by RNA within virus-infected cells, the protein kinase R (PKR) kinase phosphorylates the translation initiation factor eukaryotic initiation factor 2 (eIF2), resulting in the suppression of protein synthesis (Poppers et al., 2000). The gene products of HSV-1, specifically ICP34.5 are uniquely designed to counteract the accumulation of phosphorylated eIF2. The *US11* gene encodes an RNA-binding molecule possesses the capacity to hinder the activation of PKR (Rouse and Deshpande Kaistha, 2006). The amino acid segment of US11 comprises a recently discovered RNA-binding domain abundant in proline and basic residues, facilitating continuous protein synthesis. Owing to its RNA-binding and ribosome-binding properties, this segment alone can inhibit the activation of cellular PKR kinase in cell-free systems (Poppers et al., 2000). The protein encoded by the *US11* gene is an RNA-binding molecule that inhibits protein synthesis by blocking PKR phosphorylation of eIF2. Through its RNA-binding and ribosome-binding properties, this protein can hinder PKR kinase activation in the extracellular system. PKR is a pivotal protein kinase involved in the innate immune response of host cells. Serving as a pattern recognition receptor, PKR can detect double-stranded RNA (dsRNA), a hallmark of various viral replication processes. It is precisely this mechanism that viruses exploit to disrupt host cell immune responses.

### 4.4 ICP0

It is a multifunctional protein of significant importance in HSV-1 infection, with the *EP0* gene of PRV sharing homology (Cai et al., 2019). As widely acknowledged, pathogens undermine the immune system by employing virulence factors According to the protection hypothesis, the host has the capability to surveil (or ‘protect’) crucial innate immune pathways, activating secondary immune responses in response to disruption by virulence factors. Within human monocytes, ‘self-protection’ immune pathway operates wherein protective substances bind to a specific protein. Research has shown that ICP0, a pivotal virulence factor of HSV-1, triggers the activation of this pathway, resulting in a robust induction of the antiviral interferon IFNs (Barton et al., 2007). Surprisingly, the induction of IFNs by ICP0 occurs independently of classical immune pathways and IRF3/7 transcription factors. The ICP0-targeted protein MORC3 plays a crucial role as a negative regulator of IFN (Brukman and Enquist, 2006). ICP0 mechanistically degrades MORC3, resulting in the downregulation of the DNA element (MRE) regulated by MORC3 adjacent to the IFN locus. In addition to inhibiting MRE for the regulation of IFNB1, MORC3 also acts as a straightforward limiting factor of HSV-1. Research indicates that the principal antiviral role of MORC3 is its mechanism of self-protection, primarily accomplished through its secondary function of inhibiting IFN (Widener and Whitley, 2014). On the flip side, the virus skillfully circumvents its primary antiviral function by degrading MORC3, ultimately triggering the emergence of secondary antiviral IFN responses (Guo et al., 2009). ICP0 disrupts the host’s immune response by degrading the MORC3 protein, thereby affecting the expression and regulation of IFN. MORC3 primarily contributes to cellular immune responses, notably in antiviral immunity. Furthermore, ICP0 modulates the host’s immune response by activating specific pathways, such as the IFN pathway.

### 4.5 IE180

*IE180* and *EP0* collaborate in a superposition manner to activate both PRV gene promoters (Ou et al., 2002). The expression of *EP0* augments the infectivity of PRV genetic material. Mirroring the interaction between EP0 and IE180, the homologous counterparts ICP0 and ICP4 in HSV-1 similarly interact to boost TK gene transcription (Van Opdenbosch et al., 2012). In VZV, the presence of ORF61 and ORF62, homologous to EP0 and IE180 respectively, can also lead to the inhibition of *TK* gene transcription (Chang et al., 2004). The roles of homologous proteins in viral gene transcription may vary across different alphaherpesviruses. Although the mechanism by which EP0 and IE180 synergistically transactivate PRV gene expression remains unclear, they may interact by binding various nucleotide sequences or cellular transcription factors on the promoter (Muñoz et al., 2010).

### 4.6 ICP27

ICP27 plays a crucial role as a multifunctional protein intricately involved in various stages of viral infection. Comprising 512 amino acids, ICP27 undergoes post-translational modifications such as phosphorylation and arginine methylation, enabling it to maintain regulatory functions throughout the infection (Brideau et al., 2000). In the early stages of infection, ICP27 interacts with splicing factors, aiding in the disruption of cellular precursor mRNA splicing and playing a role in suppressing the host immune response (Newcomb et al., 2007). Additionally, in the initial phases of infection, ICP27 binds to the C-terminal domain of RNA polymerase II, directing its recruitment to the viral replication site and consequently boosting viral gene transcription. Around the 5th hour post-infection, ICP27 coordinates the generation of viral mRNA by interacting with cellular mRNA export factors 9G8, SRp20, and Aly/REF (Sandri-Goldin, 2011). At the same time, it directly associates with viral RNA via its RGG domain and engages with the cellular mRNA export receptor TAP/NXF1. Subsequently, TAP/NXF1 facilitates the export of mRNA from the nucleus via the nuclear pore complex. After being transported to the cytoplasm, ICP27 enhances the protein synthesis of viral RNA by interacting with translation initiation factors. Beyond its direct influence on mRNA processing and the expression of viral genes, ICP27 enhances viral infection by recruiting cellular molecular chaperones, particularly Hsc70, to areas enriched with molecular chaperones induced by nuclear viruses (Zhou et al., 2022). This recruitment contributes to the quality control of nuclear proteins. Furthermore, ICP27 activates NF-κB and inhibits IFN-1, subsequently suppressing cyclin 1 (STAT) phosphorylation and nuclear accumulation (Banks et al., 1993). Reports suggest that ICP27 is also implicated in suppressing activities associated with the G1 phase throughout infection and activating stress-responsive kinases. Consequently, ICP27 assumes various roles throughout the infection process, facilitated by extensive protein interactions and RNA binding. Studies have demonstrated the homology of ICP27 in every sequenced alpha herpesvirus (Kim et al., 2017). While ICP27 is the most thoroughly examined representative in this group, analyses of counterparts in other alphaherpesviruses indicate that these proteins exhibit similar activities to ICP27. Prominent examples include IE4 (VZV), UL69 (HCMV), SM (EBV), and ORF57 (Kaposi’s sarcoma-associated herpesvirus) (Ono et al., 1998). ICP27 intervenes in various cellular processes such as mRNA splicing, transcription, and translation through diverse mechanisms. Additionally, it manipulates the host’s NF-κB and IFN-1 signaling pathways to suppress the immune response.

### 4.7 US3

Initially, analysis unveiled a motif within the *US3* gene’s peptide sequence, akin to host cell protein kinases (Mori, 2012). Subsequently, US3 was identified as a crucial virulence factor in HSV-1-infected mouse. It is worth mentioning that the functionality of the US3 protein kinase is crucial for viral virulence in peripheral sites of mice, encompassing the eyes and vagina, as well as within the central nervous system (CNS) after both intracranial and peripheral infections (Wagenaar et al., 1995). US3 plays a multifaceted role by safeguarding infected cells against apoptosis, facilitating vesicle-mediated nucleocytoplasmic transit of the nucleocapsid across the nuclear envelope, modulating the morphology of the microtubule network in infected cells, evading host immune system attacks, and enhancing gene expression (Kato and Kawaguchi, 2018; Van Gent et al., 2022). This is achieved through its ability to impede histone deacetylation, stimulate mRNA translation, oversee intracellular trafficking of viruses and cellular proteins, and elevate viral enzyme levels within infected cells. This implies that US3 exhibits versatility as a protein, serving varied functions by phosphorylating multiple viral substrates across diverse processes throughout the virus lifecycle. In line with this perspective, there are reports suggesting that US3 functions as a heterologous protein kinase, potentially phosphorylating more substrates than initially anticipated. Consequently, the identification of US3 substrates is crucial for a comprehensive understanding of its function and mechanism (Graifer and Karpova, 2020). The significance of the US3 protein lies in its role in evading host immune system attacks. By shielding infected cells from apoptosis and modulating cellular processes, US3 indirectly impacts host immune responses by influencing the survival and function of immune cells. Thus, unraveling the mechanism of US3 is pivotal for comprehending how HSV-1 eludes host immune surveillance.

### 4.8 VP16

The envelope protein VP16 is pivotal in the HSV-1 virus. During viral infection, VP16 engages in physical interactions with two cytokines, creating a complex induced by VP16 (Vandevenne et al., 2010). This complex assumes a crucial function in stimulating transcription and initiating the cleavage program of early genes. VP16 interacts with a series of host factors to establish a VP16-induced regulatory switch capable of activating or deactivating the transcription of viral genes (Ding et al., 2022). Furthermore, VP16 influences various signaling pathways by associating with different host molecules intricately connected to innate immune reactions, RNA polymerases, molecular partners, and host suppression prompted by viral infections. VP16 demonstrates functional responsiveness to specific factors, such as *β*-Chain protein and PPAR-*γ*, serving as compensatory elements (Zhan et al., 2019). As a transcriptional activator, VP16 is involved in controlling neuronal lytic infection and latency reactivation. The VP16 promoter features distinctive neural-specific sequences crucial for reactivating neurons during latency. Importantly, these sequences can be activated by factors linked to neurons, as well as by viral DNA replication (Yu et al., 2018). While the examination of neuronal-related factors remains incomplete, significant advancements have been achieved regarding host factors associated with VP16. Incorporating *β*-chain proteins, lamin A/C, and HCF-1, several cytokines have been confirmed to interact directly or indirectly with VP16, thereby regulating viral infection through multiple mechanisms. Additionally, these associations could be jointly controlled by viral factors like ICP22 and ICP0 (Granzow et al., 2001). The interaction between VP16 and various host factors influences diverse signaling pathways, encompassing those pertinent to innate immune responses, RNA polymerases, and molecular chaperones, among others. Additionally, VP16, functioning as a transcriptional activator, contributes to neuronal lytic infection and latency reactivation. Notably, its promoter harbors specific sequences associated with neurons, which can be activated by neuronal-related factors and viral DNA replication. These findings collectively underscore the indispensable role of VP16 in immune evasion.

### 4.9 UL31

The PRV UL31 protein shares homology with the HSV-1 UL31 late protein and consists of 271 amino acid residues (Li et al., 2014). UL31 assumes a crucial function in viral replication through its association with the nuclear matrix and facilitation of the assembly of the viral nuclear export complex (Zhu et al., 1999). Its function is exerted through the suppression of the retinoic acid-inducible gene I receptor pathway mediated IFN-*β* response, thereby inhibiting the activation of IFN-*β* production and subsequent expression of downstream interferon-stimulated genes (ISGs) (Li et al., 2015). Furthermore, UL31 impedes IFN-*β* activation by targeting IRF3/IRF7 signaling pathways. Mechanistically, UL31 interacts with key proteins such as TANK-binding kinase 1 (TBK1), inducible IκB kinase (IKK*i*), and IRF3, hampering the assembly of IKK*i*-IRF3 complexes and obstructing the association and nuclear migration of IRF3 (Gong et al., 2022). The activation of RIG-I plays a pivotal role in promoting dendritic cell maturation and antigen presentation, essential for T cell activation and the initiation of adaptive immune responses. Moreover, activation of the RIG-I signaling pathway can influence the functions of T cells and natural killer (NK) cells, thereby bolstering the cellular immune response. The PRV UL31 protein inhibits IFN-*β* production mediated by the RIG-I receptor pathway, thus suppressing the expression of interferon and downstream interferon-stimulated genes (ISGs). Additionally, UL31 disrupts IFN-*β* activation by interfering with the IRF3/IRF7 signaling pathway.

### 4.10 UL34

The *UL34* gene product functions as a phosphorylated protein linked to the nuclear membrane, acting as a target for the PRV US3 encoded kinase (Zhang et al., 2023). The UL34 and UL31 proteins play crucial roles at the onset of herpesvirus nucleocapsid budding. They establish a complex situated at the nuclear periphery, enabling either the maturation or export of viral particles from infected cells. It has been shown the positioning of HSV-1 US3, UL31, and UL34 proteins within the host cells (Liu et al., 2014). UL31 and UL34 proteins display adherence to perinuclear virions while staying unattached to extracellular virions. HSV-1 UL34, UL31, and US3 proteins assemble into a complex that interacts with UL47. This intricate structure localizes at the nuclear membrane of infected cells and is essential for facilitating viral nuclear export (Fuchs et al., 2002).

### 4.11 UL36

Reports have revealed that the interplay between the viral envelope and the particle shell prominently features the largest protein identified in the herpes virus (Cardone et al., 2012). It has been widely reported in studies on both PRV and HSV-1. The UL36 proteins have been proposed to participate in direct physical interactions with pivotal granule proteins. The absence of the UL36 proteins results in the accumulation of uncoated HSV-1 virus particles within the cytoplasm. Simultaneously, the absence of the UL37 protein is associated with an augmented presence of uncoated HSV-1 particles in the cytoplasm, representing another essential coating component. Researchers have recently demonstrated that cells infected with PRV *UL37* deletion mutants exhibit organized particle aggregates within the cytoplasm (Klupp et al., 2001). The particles do not appear to make direct contact; they interact through extended connections emanating from the vertices of the particles. Deposition of the *UL36* gene product onto the particles is anticipated, and the subsequent encapsulation process may be impeded in the absence of the UL37 protein. Hence, the aggregation may result from anomalous UL36-UL36 interactions (Collantes et al., 2022). Under these circumstances, the conceivable physical interaction between UL37 and UL36 proteins represents a vital initial stage in the encapsulation process. Following investigation, the PRV UL36 protein was identified, and it was noted that without UL37, the protein exists in granular aggregates within the cytoplasm but goes unnoticed in the initial encapsulated granules near the nucleus (Klupp et al., 2002). Furthermore, researchers verified that UL36 and UL37 proteins indeed undergo physical interactions. The membrane proteins UL36 and UL37 of PRV demonstrate direct physical interactions. Remarkably, even with the absence of the UL37, UL36 remains discernible on the cytoplasmic inner shell. Nonetheless, the deficiency of PRV or HSV-1 UL37 protein, along with HSV-1 UL36 protein, can impede or prevent the subsequent elongation and encapsulation processes (Fuchs et al., 2004). Thus, posit that the initiation of cytoplasmic capsid formation involves the interaction between the UL36 protein and the capsid, succeeded by the interaction between the UL37 and UL36 proteins (Shanda and Wilson, 2008). Through this sequence, the ordered addition of the innermost layer to the newly formed virus particle envelope becomes achievable. Additional protein–protein interactions and the involvement of non-conserved coating components could elucidate the comprehensive functional assembly of the coating. Ultimately, the engagement amidst the envelope protein alongside the carboxyl end of the viral protein within the trans-Golgi vesicle may catalyze the completion of the final envelope (Jambunathan et al., 2014). In summary, UL36 and UL37 proteins exhibit physical interaction, and UL36 can remain localized within the cytoplasm even in the absence of the UL37 protein. However, the absence of either protein may impede or halt subsequent elongation and encapsulation processes. The interaction between UL36 protein and viral particles facilitates the systematic addition of newly formed virus particle envelopes. Furthermore, the engagement between the carboxyl end of the viral envelope protein and the viral protein within the trans-Golgi vesicle may catalyze the finalization of the envelope assembly.

## 5 Conclusion

Currently, the research and development of vaccines targeting alphaherpesviruses are still in the developmental phase. Taking PRV as an example, despite the successful eradication of the disease from commercial pig herds in some countries through the utilization of gene deletion vaccines and strategies such as differentiation of infected from vaccinated animals (DIVA) (Lei et al., 2016), PRV remains a significant concern in numerous countries, especially those with dense pig populations. Despite the widespread adoption of gene deletion vaccines and regional eradication initiatives utilizing DIVA (Wang et al., 2014), PRV persists as an endemic disease in most Chinese provinces, primarily due to insufficient mandatory vaccination campaigns, inadequate awareness, and biosecurity measures (Sun et al., 2016). Currently, no safe and effective vaccines have been produced for feline herpesvirus, bovine herpesvirus, or caprine herpesvirus (Gomes Noll et al., 2019; Kim et al., 2024). Only some feasible solutions are still under development (Jiao et al., 2024). Hence, during this phase when vaccine development remains incomplete, investigating the immunoescape mechanism of alphaherpesviruses becomes particularly imperative.

In this review, we elucidate the precise mechanisms by which alphaherpesviruses establish dormant infections in neurons and propagate through the neural network. These viruses possess inherent characteristics that allow them to adeptly evade immune detection, earning them the title of immunoescape artists within the herpesvirus family. Given their propensity for long-term latent carriage and lifelong viral presence, efforts to counteract immunoescape become imperative. Achieving virus purification necessitates a deep understanding of these evasion strategies. Therefore, comprehending the intricacies of viral immunoescape mechanisms is paramount for effective control and containment. Consequently, designing targeted interference methods to combat viral immunoescape and unraveling the viral potential life cycle are critical pursuits.

Insights gleaned from investigating the immunoescape strategies utilized by alphaherpesviruses can enhance our comprehension of the intricate tactics employed by these viruses to elude and manipulate host immune defenses. This understanding is pivotal in advancing the development of potent antiviral strategies, vaccines, and therapeutics aimed at combatting alphaherpesvirus infections.

The immune escape process of the virus is crucial for the survival of the alphaherpesvirus. VZV presents clinically as herpes zoster and causes postherpetic neuralgia (PHN) and a series of complications. And under the condition of immune escape, it carries the virus for life and relapses. Therefore, promoting the study of the functional domains of various viral genes and the interactions between viral proteins and host proteins in clinical practice is crucial for the prevention and treatment of herpes simplex virus infection. By studying various functional domains, we can better target specific functional domains and thus block the immune escape process. And further exploration of the virus lifecycle process and potential mechanisms will make a significant contribution to our goals. Research on immunoescape mechanisms has unveiled various viral proteins or genetic components, such as ICP34.5 and US11 in the context of HSV-1 (Singh and Tscharke, 2020), and UL36 and UL37 in the context of PRV (Cui et al., 2023), which play pivotal roles in facilitating the immunoescape of host immune responses. Understanding the interactions at the molecular level between viral elements and host immune components provides valuable insights that can guide the development of targeted interventions aimed at effectively disrupting these strategies of immunoescape.

Moreover, a profound comprehension of the immunoescape strategies employed by alphaherpesviruses enhances our insight into viral pathogenesis and the intricate dynamics of host-virus interactions. Leveraging these advancements in research can be instrumental in designing vaccines capable of eliciting robust and targeted immune responses, while also advancing tailored antiviral drugs to disrupt viral immunoescape mechanisms with precision.

## Author contributions

YW: Writing – original draft, Writing – review & editing, Conceptualization. CM: Writing – original draft, Writing – review & editing. SW: Writing – review & editing. HW: Writing – review & editing. XC: Writing – review & editing. JM: Writing – review & editing. LW: Writing – review & editing, Funding acquisition, Writing – original draft. H-JQ: Writing – review & editing, Conceptualization, Supervision, Writing – original draft. YS: Writing – review & editing, Conceptualization, Supervision, Writing – original draft.
